# Physicochemical and Mechanical Characterization of Silk and PGLA Dental Sutures Following Humic-Acid-Containing Surface Treatment

**DOI:** 10.3390/polym18212676

**Published:** 2026-10-28

**Authors:** Aysun Akpınar, Halil Çelik, Mehmet Kılınç, Zeynep Ceren Çelik, Ali Merdji, Sandipan Roy

**Affiliations:** 1 Department of Periodontology, Faculty of Dentistry, Bursa Uludag University, Bursa 16120, Turkey; 2 Department of Oral and Dental Health, Vocational School of HealthCare Services, Istinye University, Istanbul 34396, Turkey; halil.celik@istinye.edu.tr; 3 Department of Textile and Fashion, Şebinkarahisar School of Applied Sciences, Giresun University, Giresun 28400, Turkey; 4 Department of Restorative Dentistry, Faculty of Dentistry, Bursa Uludag University, Bursa 16120, Turkey; 5 Department of Mechanical Engineering, Faculty of Science & Technology, University Mustapha Stambouli of Mascara, Mascara 29000, Algeria; 6 Laboratory of Mechanics Physics of Materials (LMPM), Faculty of Technology, University Djillali Liabés of Sidi Bel-Abbés, Sidi Bel-Abbès 22000, Algeria; 7 Department of Mechanical Engineering, SRM Institute of Science and Technology, Kattankulathur 603203, India

**Keywords:** surgical sutures, humic acid, silk, poly(glycolide-co-lactide) (PGLA), surface coating, FTIR spectroscopy, tensile properties

## Abstract

Surface coating may influence the physicochemical and mechanical properties of surgical sutures. This study was conducted to characterize the changes associated with a humic-acid-containing alginate coating applied to silk and poly(glycolide-co-lactide) (PGLA) sutures in two sizes (3-0 and 4-0). Untreated sutures served as controls. Surface-related spectral changes were examined using Fourier-transform infrared (FTIR) spectroscopy, while mechanical properties were evaluated via tensile testing in accordance with EN ISO 2062. Maximum tensile force and elongation at break were recorded. FTIR analysis revealed changes in the intensity and breadth of selected absorption bands, particularly those associated with O–H and C=O groups, after the coating was applied. These findings indicated treatment-associated spectral changes but did not independently confirm humic acid incorporation or coating stability. The maximum tensile force was lower in treated silk sutures than in untreated controls for both size 3-0 (22.6 ± 3.68 N vs. 17.8 ± 1.35 N; *p* = 0.140) and size 4-0 (12.8 ± 1.21 N vs. 10.6 ± 2.56 N; *p* = 0.276), although neither difference was statistically significant. For PGLA sutures, no statistically significant difference was observed for size 3-0 (27.7 ± 2.80 N vs. 27.6 ± 2.56 N; *p* = 0.966). In contrast, the size 4-0 PGLA sutures exhibited a higher maximum tensile force following treatment (14.2 ± 1.85 N vs. 21.1 ± 2.45 N; *p* = 0.021). Elongation at break increased in the treated size 3-0 silk sutures (16.0 ± 0.91% vs. 20.3 ± 1.10%; *p* = 0.007) and decreased in the treated size 4-0 silk sutures (19.3 ± 1.26% vs. 14.8 ± 1.75%; *p* = 0.027). No statistically significant differences in elongation were found for size 3-0 PGLA (32.1 ± 1.16% vs. 28.4 ± 2.21%; *p* = 0.082) or size 4-0 PGLA sutures (31.4 ± 1.88% vs. 30.8 ± 1.24%; *p* = 0.672). These preliminary findings indicate that the humic-acid-containing alginate coating procedure was associated with spectral changes and different mechanical responses in silk and PGLA sutures.

## 1. Introduction

Surgical sutures function not only as mechanical closure devices but also as temporary biomaterial interfaces that directly interact with biological tissues during wound healing [1,2]. Their performance depends on a delicate balance between structural stability, degradation behavior, and surface chemistry. In oral and dental surgery, where microbial exposure and inflammatory responses are pronounced, the physicochemical characteristics of suture materials play a decisive role in clinical outcomes. Suture materials vary according to origin, structural configuration, and degradation profile.

Natural fibers such as silk are widely used due to their favorable handling properties and knot security; however, they are non-absorbable and typically require postoperative removal [3]. In contrast, synthetic polymers, including polyglycolic acid (PGA) and poly(glycolide-co-lactide) (PGLA), undergo progressive hydrolytic degradation, allowing gradual resorption without secondary intervention [4]. Structural design further influences clinical behavior: monofilament sutures generally induce less tissue trauma, whereas braided sutures provide improved flexibility and knot stability [5]. Therefore, suture selection depends on surgical location, healing duration, and infection risk [6]. Beyond their mechanical role, sutures have recently been explored as platforms for therapeutic delivery. The incorporation of antimicrobial agents, essential oils, or anti-inflammatory compounds has been proposed to enhance wound healing and reduce microbial colonization [7,8,9]. Such strategies aim to transform sutures from passive closure materials into bioactive systems capable of locally modulating the wound microenvironment. Humic acid, a naturally occurring organic macromolecular complex derived from the decomposition of plant matter, has attracted increasing interest due to its antimicrobial, antioxidant, and anti-inflammatory properties [10,11]. However, these properties may vary according to their source, chemical composition, purification, concentration, and experimental conditions. Therefore, findings reported for humic substances in other formulations or experimental systems cannot be directly extrapolated to the specific commercial humic acid preparation used in this study. Chemically, humic acid consists of heterogeneous aromatic and aliphatic structures enriched with carboxyl (-COOH), hydroxyl (-OH), and phenolic functional groups [12]. These chemical moieties confer strong chelating capacity and redox activity while also enabling intermolecular interactions with polymeric substrates. Owing to its reported biocompatibility and ability to influence inflammatory pathways, humic acid has been proposed for intraoral biomedical applications, including dental formulations and functionalized biomaterials such as sutures [13].

However, the reported biological properties of humic acid alone do not establish its suitability as a suture coating or surface functionalization agent. A critical unresolved issue is whether it is possible to incorporate humic acid without adversely affecting the mechanical properties of the underlying suture material. Moreover, the response to surface functionalization may differ substantially between protein-based natural fibers such as silk and synthetic polyester-based materials such as PGLA. There is still little comparative data describing these material-dependent effects, particularly across clinically relevant suture calibers.

Successful incorporation of bioactive compounds into suture substrates necessitates preserving the carrier material’s mechanical integrity. Alterations in surface chemistry must not compromise tensile strength or structural stability, as these properties are critical for maintaining wound approximation during early healing phases [5,14,15]. Furthermore, confirming the presence of the bioactive agent on the material surface and evaluating its interaction with the polymer matrix are essential steps in validating functionalization strategies [16]. Analytical techniques such as Fourier-transform infrared (FTIR) spectroscopy enable identification of chemical modifications through shifts in characteristic functional groups, while mechanical testing provides a quantitative assessment of tensile strength and elongation behavior [17]. The combined use of these methods facilitates comprehensive evaluation of structure–property relationships following surface treatment.

Despite growing interest in bioactive surgical sutures, the surface functionalization of clinically relevant dental suture materials with humic acid remains insufficiently characterized, particularly with respect to the relationship between surface chemical modification and mechanical performance. This study provides a comparative evaluation of humic acid functionalization in both natural silk and synthetic poly(glycolide-co-lactide) (PGLA) dental sutures across two clinically relevant calibers (3-0 and 4-0). The novelty of this study lies in the direct comparison of material- and caliber-dependent responses to the same surface treatment, combining FTIR-based chemical characterization with quantitative tensile testing and elongation analysis. This approach is used to determine whether humic acid functionalization can modify the suture surface while preserving the mechanical properties required for clinical handling. The findings therefore provide structure–property insight for the development of humic-acid-functionalized surgical sutures as potential bioactive biomaterial platforms.

This study was conducted to characterize treatment-associated spectral changes and evaluate the tensile behavior of silk and poly(glycolide-co-lactide) (PGLA) dental sutures in two sizes (3-0 and 4-0) after coating with a humic-acid-containing alginate. Untreated sutures were used as controls. FTIR spectroscopy was employed to identify changes in characteristic absorption bands, while tensile testing was used to assess the maximum tensile force and elongation at break. This study was designed as a preliminary physicochemical and mechanical investigation and was not intended to establish chemical bonding, coating stability, biological efficacy, or mechanical equivalence between treated and untreated sutures.

## 2. Materials

Four independent suture specimens were prepared for each experimental group (*n* = 4). Of these, three specimens were used for mechanical testing (*n* = 3), while one independent specimen was used for FTIR characterization. Thus, the experimental groups consisted of four independently prepared specimens, with separate specimens allocated to mechanical and spectroscopic analyses. Following preparation and air-drying, the specimens were stored in a closed, airtight container under dry conditions until mechanical testing and FTIR analysis. The specimens were handled carefully to minimize environmental exposure before testing.

### 2.1. Preparation of Humic-Acid-Impregnated Sutures

Commercially available multifilament absorbable poly(glycolide-co-lactide) (PGLA) (Pegelak, Dogsan, Trabzon, Türkiye) and non-absorbable multifilament silk sutures (Trusilk, Healthium Medtech, India) in sizes 3-0 and 4-0 were used. Humic acid (Humic acid pure 99%, Serravit^®^, Kocaeli, Türkiye) was incorporated into the suture surface using a sodium alginate-based coating solution under controlled conditions. Briefly, 1.87 g of sodium alginate was dissolved in 125 mL of distilled water under magnetic stirring at 1600 rpm and 100 °C for 100 min to prepare the stock alginate solution. Subsequently, 0.05 mL of humic acid was added to 10 mL of the alginate solution and stirred at 500 rpm for 15 min. The sutures were immersed in the humic acid–alginate mixture for 1 min. Following treatment, the sutures were transferred into a 100 mL aqueous bath containing 2.21 g CaCl_2_ and maintained for 5 min to induce gelation of the coating. The treated sutures were subsequently air-dried for 48 h and stored under sterile conditions until analysis. Four independent suture specimens were prepared for each experimental group.

### 2.2. Fourier-Transform Infrared (FTIR) Spectroscopy

FTIR spectroscopy was used to characterize chemical and surface modifications associated with the humic acid treatment. Both treated and untreated silk and PGLA sutures in 3-0 and 4-0 sizes were analyzed. Measurements were performed using an attenuated total reflectance (ATR) accessory over a spectral range of 4000–500 cm^−1^ at a resolution of 4 cm^−1^. Before analysis, the samples were gently rinsed with distilled water and air-dried. Each specimen was placed in direct contact with the diamond crystal, using uniform pressure to ensure consistent surface contact. Background spectra were collected before each measurement, and 32 scans were averaged for each specimen to improve the signal-to-noise ratio. Characteristic absorption bands were analyzed, including O–H/N–H stretching (~3300 cm^−1^), C–H stretching (~2920 cm^−1^), C=O stretching (~1700–1750 cm^−1^), and ester or ether vibrations (1200–1000 cm^−1^). Shifts in peak positions, broadening, or intensity variations were interpreted as indicative of humic acid binding to the suture surface. Data were processed and visualized using dedicated spectral analysis software.

### 2.3. Tensile Testing

Tensile testing was performed using a universal testing machine (AG-X 5 kN, Shimadzu Corporation, Kyoto, Japan) equipped with a 5 kN load cell, in accordance with EN ISO 2062 (International Organization for Standardization: Geneva, Switzerland, 2009). Untreated and humic-acid-treated sutures were tested in triplicate (*n* = 3 per group) at a crosshead speed of 250 mm/min and a gauge length of 250 mm under controlled environmental conditions (20 ± 2 °C and 65 ± 4% relative humidity). The maximum tensile force and elongation at break were recorded for each specimen. The results are expressed as the mean ± standard deviation (SD). Statistical analyses were performed using Microsoft Excel, with *p* < 0.05 considered statistically significant.

## 3. Results and Discussion

**Figure 1:**
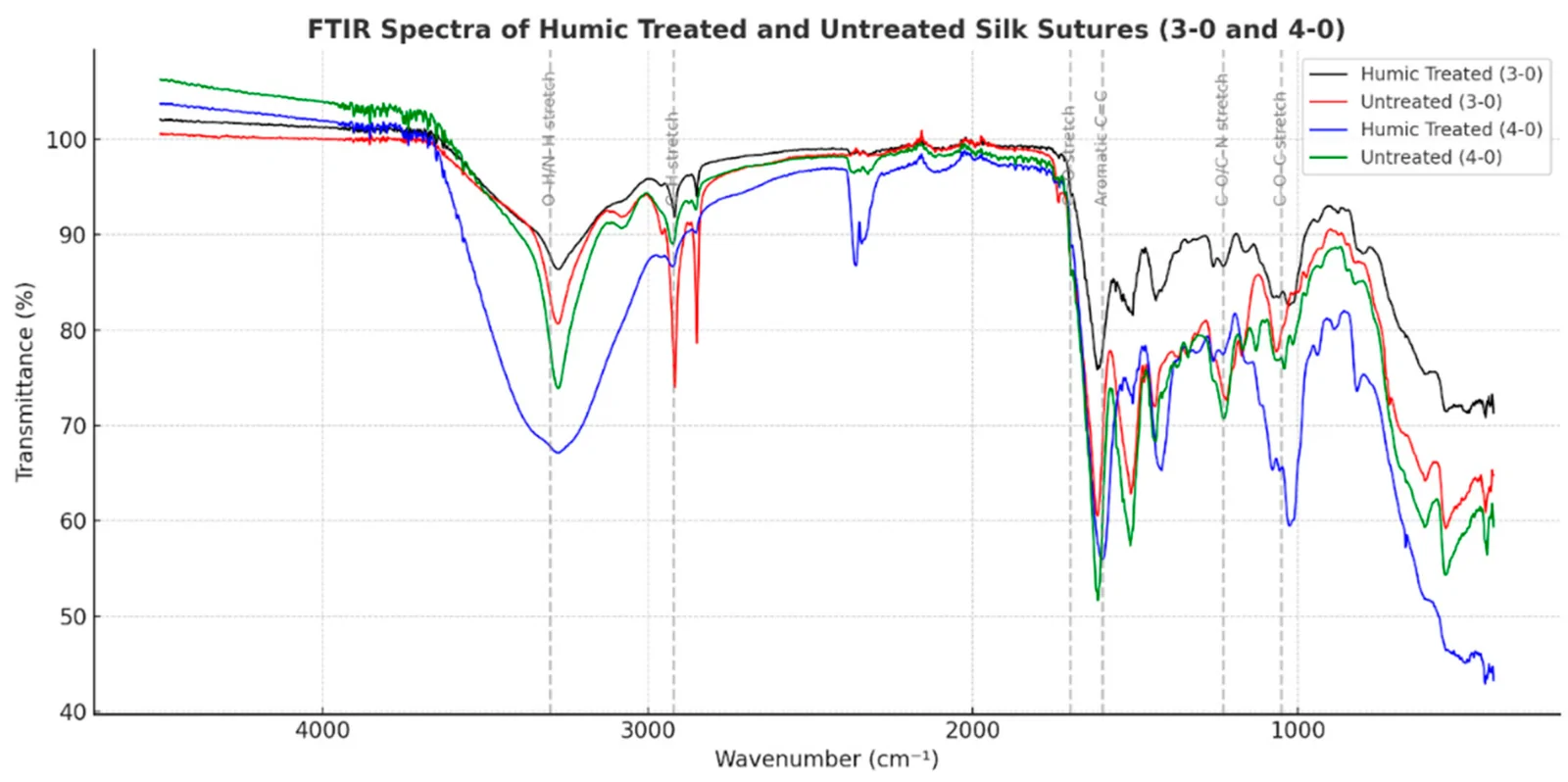
Comparative FTIR spectra of untreated and humic-acid-treated silk sutures (3-0). Key absorption bands corresponding to functional groups are indicated: O–H/N–H stretching (~3300 cm^−1^), C–H stretching (~2920 cm^−1^), C=O stretching (~1700 cm^−1^), aromatic C=C vibrations (~1600 cm^−1^), and C–O/C–N stretching (~1230 cm^−1^).

**Figure 2:**
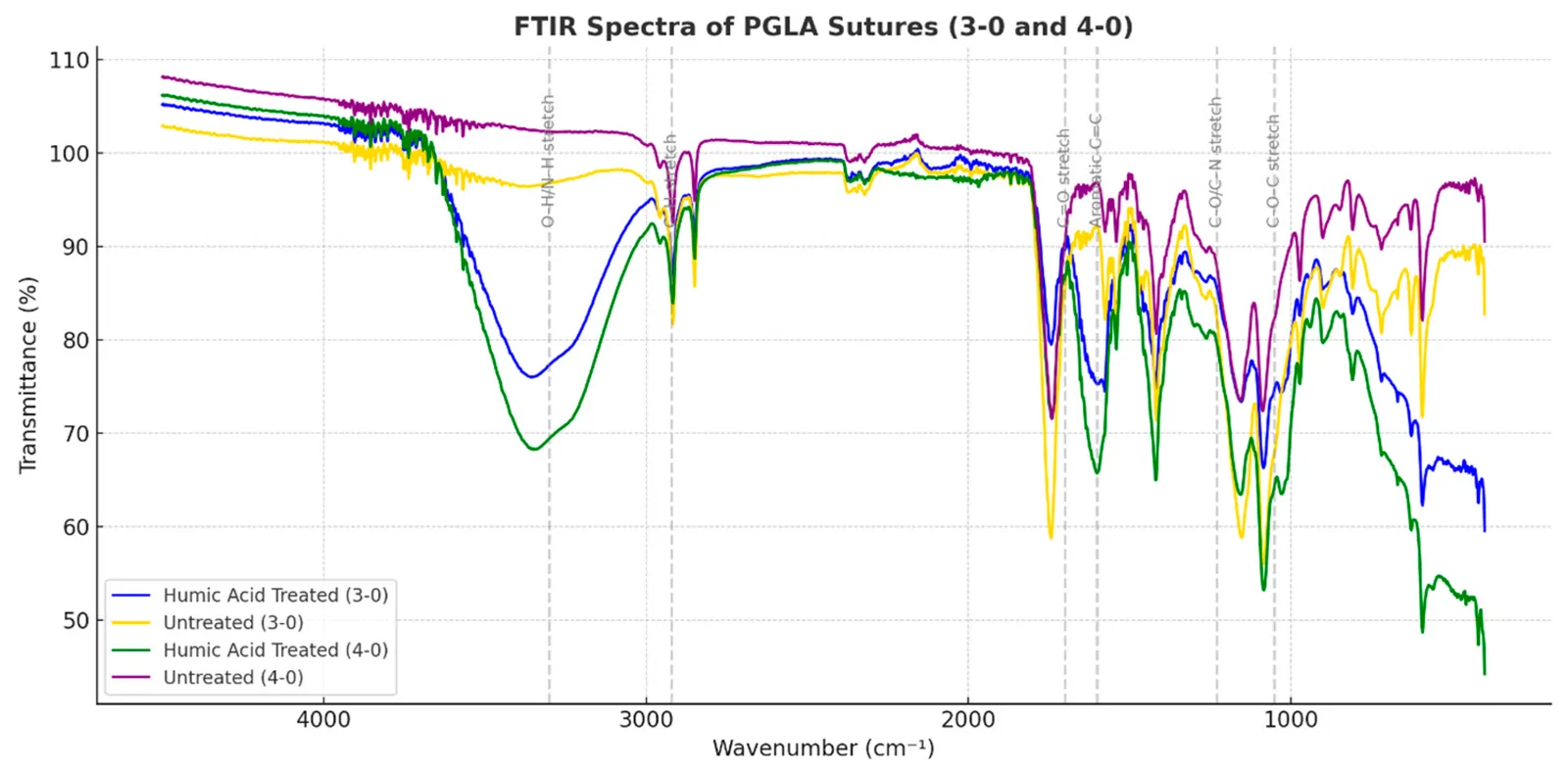
Comparative FTIR spectra of untreated and humic-acid-treated PGLA sutures (3-0). Key absorption bands corresponding to functional groups are indicated: O–H/N–H stretching (~3300 cm^−1^), C–H stretching (~2920 cm^−1^), C=O stretching (~1700 cm^−1^), aromatic C=C vibrations (~1600 cm^−1^), and C–O/C–N stretching (~1230 cm^−1^).

FTIR spectroscopy revealed distinct chemical modifications in the humic-acid-treated silk and PGLA sutures compared with the untreated controls. The silk sutures (3-0 and 4-0) exhibited the characteristic absorption bands of silk fibroin, including broad O–H/N–H stretching (~3300 cm^−1^), C–H stretching (~2920 cm^−1^), C=O stretching (~1700 cm^−1^), aromatic C=C and amide II (~1600 cm^−1^), and C–O/C–N stretching in the 1230–1050 cm^−1^ region. The humic-acid-treated silk sutures displayed increased peak intensity and broadening, particularly in the O–H and C=O regions, indicating successful adsorption of humic acid and incorporation of additional hydroxy and carboxyl functional groups. These spectral changes were more pronounced in the 4-0 samples, suggesting that suture diameter or surface exposure may influence the extent of humic acid interaction (Figure 1 and Figure 2). PGLA sutures exhibited characteristic polymeric absorption bands, including O–H/N–H stretching (~3300 cm^−1^), C–H stretching (~2920 cm^−1^), a strong carbonyl (C=O) peak (~1750–1700 cm^−1^), and ester-related C–O–C stretching (1200–1000 cm^−1^). The humic-acid-treated PGLA sutures demonstrated broader and more intense O–H and C=O bands, consistent with successful surface functionalization. Subtle spectral shifts and intensity variations between the 3-0 and 4-0 PGLA samples may reflect differences in surface area or diameter affecting humic acid adsorption. The untreated sutures retained sharp, well-defined peaks corresponding to the native polymer structure. Overall, FTIR analysis confirmed effective surface functionalization of both silk and PGLA sutures.

### Tensile Strength and Elongation

Mechanical testing revealed different responses to the humic-acid-containing alginate coating procedure in the silk and PGLA sutures (Table 1 and Table 2). In the silk sutures, the maximum tensile force decreased from 22.6 ± 3.68 N to 17.8 ± 1.35 N in the size 3-0 sutures (*p* = 0.140) and from 12.8 ± 1.21 N to 10.6 ± 2.56 N in the size 4-0 sutures (*p* = 0.276). However, neither difference was statistically significant. For the PGLA sutures, the maximum tensile force remained similar after treatment for the size 3-0 sutures (27.7 ± 2.80 N untreated vs. 27.6 ± 2.56 N treated, *p* = 0.966), whereas the size 4-0 sutures showed a significant increase from 14.2 ± 1.85 N to 21.1 ± 2.45 N (*p* = 0.021).

Elongation at break also varied according to suture material and size. In the silk sutures, elongation increased from 16.0 ± 0.91% to 20.3 ± 1.10% in size 3-0 (*p* = 0.007) and decreased from 19.3 ± 1.26% to 14.8 ± 1.75% in size 4-0 (*p* = 0.027); both differences were statistically significant. In the PGLA sutures, elongation decreased from 32.1 ± 1.16% to 28.4 ± 2.21% in the size 3-0 sutures (*p* = 0.082) and from 31.4 ± 1.88% to 30.8 ± 1.24% in the size 4-0 sutures (*p* = 0.672), although neither difference reached statistical significance. Overall, the coating procedure was associated with changes in tensile behavior and elongation that varied by suture material and size. These findings should be interpreted cautiously given the small sample size and the absence of an alginate-only control.

In this study, the physicochemical and mechanical effects of humic acid treatment on non-absorbable silk and absorbable PGLA sutures of two sizes (3-0 and 4-0) were evaluated. The percentage-change analysis highlights the material-dependent response to humic acid functionalization. The silk sutures demonstrated a measurable reduction in tensile strength following treatment, whereas the PGLA sutures showed almost complete preservation of tensile strength. This difference may be related to the different chemical and structural characteristics of the two substrates. Silk is a protein-based natural fiber whose mechanical properties are closely associated with its fibroin structure and intermolecular interactions. Surface exposure to the humic-acid–alginate coating environment may therefore alter surface interactions and contribute to changes in tensile behavior. In contrast, PGLA is a synthetic polyester with a relatively stable polymeric structure, which may limit the extent to which the surface treatment affects its bulk mechanical properties. The relatively small changes observed in PGLA’s tensile strength, particularly in the 3-0 specimens (−0.4%), support the interpretation that the treatment predominantly affected the surface rather than substantially altering the bulk polymer structure. These findings suggest that the underlying suture material is an important determinant of the mechanical outcome of humic acid functionalization. FTIR spectra revealed treatment-associated changes in selected absorption bands, while mechanical testing indicated preserved structural integrity, indicating potential clinical advantages in tissue compliance and reduced infection risk. For silk sutures, untreated samples exhibited characteristic fibroin peaks, including O–H/N–H stretching (~3300 cm^−1^), C–H stretching (~2920 cm^−1^), C=O stretching (~1700 cm^−1^), aromatic C=C and amide II vibrations (~1600 cm^−1^), and C–O/C–N stretching (1230–1050 cm^−1^). Humic-acid-treated silk showed increased intensity and broadening in the O–H/N–H and C=O regions, indicating adsorption of humic acid and introduction of additional hydroxyl and carboxyl groups, likely via hydrogen bonding and van der Waals interactions [18,19]. These chemical modifications may enhance the bioactive properties of the sutures, potentially contributing to their anti-inflammatory and antimicrobial effects [20,21,22]. The effect was more pronounced in the 4-0 sutures, suggesting that suture diameter and surface area influence functionalization efficiency.

Mechanical testing revealed a moderate reduction in tensile strength in the silk sutures after humic acid treatment (e.g., 4-0: 12.8 N → 10.6 N), while their elongation at break increased, particularly in the 3-0 samples. This behavior likely reflects the hydrophilic and protein-based nature of silk fibers, which are more sensitive to surface modifications. Humic acid impregnation may alter fiber–matrix interactions or introduce additional surface porosity, slightly reducing load-bearing capacity. Clinically, these findings suggest that treated silk sutures retain adequate handling properties but may require caution in high-tension applications. In contrast, the PGLA sutures maintained both tensile strength and elongation after humic acid treatment, demonstrating that the synthetic polyester structure resists surface-induced mechanical compromise [9,23,24]. FTIR spectra showed treatment-associated changes in selected absorption bands without penetration of the polymer core, consistent with the inherent hydrolytic stability and crystallinity of PGLA. The PGLA sutures showed no statistically significant changes in maximum tensile force following the coating procedure; however, mechanical equivalence was not established.

Overall, the humic-acid-containing alginate coating procedure was associated with spectral changes in both silk and PGLA sutures while preserving clinically relevant mechanical performance, with the magnitude of mechanical impact dependent on both material type and suture diameter. FTIR analysis further revealed characteristic peaks corresponding to carboxylic, phenolic, and hydroxyl groups, with amide band shifts suggesting physical or chemical interactions between humic acid and the polymer matrix [25,26]. The physicochemical and mechanical behavior of surface-modified dental materials depends on the interaction between the treatment procedure, the characteristics of the underlying substrate, and the environmental conditions to which the material is exposed. Aliberti et al. (2025) demonstrated that pH, temperature, and exposure time significantly influenced the release of multiple ions from bioactive restorative materials, emphasizing that material properties and responses may vary under different environmental conditions [27]. Although their study investigated ion release rather than mechanical properties or surgical sutures, it highlights the importance of evaluating dental materials under conditions relevant to their intended application. Similarly, Aliberti et al. (2026) emphasized that simplified material models, idealized bonding conditions, and static loading protocols can limit the clinical interpretation of finite element analysis findings in polymer-based adhesive restorations [28]. Although this review focused on computational modeling rather than experimental tensile testing, it reinforces the need to interpret material performance predictions within the limitations of the methods employed. These considerations are particularly relevant to the present study, in which a humic-acid-containing alginate coating procedure was associated with different mechanical responses in silk and poly(glycolide-co-lactide) (PGLA) sutures. These findings indicate that humic-acid-impregnated sutures have potential as multifunctional surgical materials, as they combine mechanical reliability with antimicrobial and anti-inflammatory capabilities. Future studies should investigate cytocompatibility, antibacterial efficacy, degradation behavior, and in vivo performance to fully elucidate their clinical applicability.

This study has several limitations. First, the small sample size for mechanical testing (n = 3 per experimental group) may limit statistical power and generalizability; therefore, the findings should be confirmed in larger studies with appropriate power calculations. Second, antimicrobial activity and biological safety, including cytotoxicity, were not evaluated. Thus, the reported physicochemical and mechanical findings cannot establish antimicrobial efficacy, biocompatibility, or clinical safety. Third, the degradation behavior of the humic-acid-treated PGLA sutures remains unknown and requires further investigation. Fourth, the absence of a matrix-only control group (sodium alginate/CaCl_2_ without humic acid) prevents independent assessment of the effects of humic acid and the coating matrix. In addition, coating thickness, uniformity, and surface morphology were not directly characterized, as FTIR analysis alone cannot establish coating continuity or structural integrity. Finally, the mechanisms underlying the reduction in maximum tensile force observed in the coated silk sutures could not be determined. Further studies incorporating appropriate controls, surface and structural characterization, degradation analysis, and biological testing are needed to address these limitations and establish the suitability of the modified sutures for biomedical applications.

## 4. Conclusions

Humic-acid-impregnated sutures demonstrate effective surface functionalization while maintaining clinically relevant mechanical properties. Silk and PGLA sutures retained sufficient tensile strength and flexibility, with PGLA showing superior structural stability after treatment These chemical modifications may enhance the bioactive properties of the sutures, potentially contributing to their anti-inflammatory and antimicrobial effects. Overall, humic-acid-functionalized sutures combine mechanical reliability with enhanced bioactivity, supporting their prospective use in clinical surgical settings.

## Figures and Tables

**Table 1:** Maximum tensile force (N) of dental surgical sutures before and after humic acid impregnation.

Suture Sample	Maximum Force (N) Mean ± SD	Min–Max (N)	*p*
Silk 3-0 untreated	22.6 ± 3.68	18.7–26.0	0.140
Silk 3-0 humic-acid-impregnated	17.8 ± 1.35	16.5–19.2	
Silk 4-0 untreated	12.8 ± 1.21	11.5–13.9	0.276
Silk 4-0 humic-acid-impregnated	10.6 ± 2.56	7.9–13.0	
PGLA 3-0 untreated	27.7 ± 2.80	24.9–30.5	0.966
PGLA 3-0 humic-acid-impregnated	27.6 ± 2.56	24.9–30.0	
PGLA 4-0 untreated	14.2 ± 1.85	12.3–16.0	0.021
PGLA 4-0 humic-acid-impregnated	21.1 ± 2.45	18.7–23.6	

**Table 2:** Elongation at break (%) of dental surgical sutures before and after humic acid impregnation.

Suture Sample	Elongation (%), Mean ± SD	Min–Max (%)	*p* Value
Silk 3-0 untreated	16.0 ± 0.91	14.8–17.0	0.007
Silk 3-0 humic-acid-impregnated	20.3 ± 1.10	18.9–21.6	
Silk 4-0 untreated	19.3 ± 1.26	18.08–21.04	0.027
Silk 4-0 humic-acid-impregnated	14.8 ± 1.75	12.37–16.45	
PGLA 3-0 untreated	32.1 ± 1.16	31.14–33.73	0.082
PGLA 3-0 humic-acid-impregnated	28.4 ± 2.21	25.33–30.47	
PGLA 4-0 untreated	31.4 ± 1.88	28.78–33.07	0.672
PGLA 4-0 humic-acid-impregnated	30.8 ± 1.24	29.08–31.98	

## Data Availability

The original contributions presented in this study are included in the article. Further inquiries can be directed to the corresponding author.
